# Population structure and diversity of the needle pathogen *Dothistroma pini* suggests human-mediated movement in Europe

**DOI:** 10.3389/fgene.2023.1103331

**Published:** 2023-02-16

**Authors:** Ariska van der Nest, Michael J. Wingfield, Dušan Sadiković, Martin S. Mullett, Benoit Marçais, Valentin Queloz, Katarina Adamčíková, Kateryna Davydenko, Irene Barnes

**Affiliations:** ^1^ Department of Biochemistry, Genetics and Microbiology, Forestry and Agricultural Biotechnology Institute (FABI), University of Pretoria, Pretoria, South Africa; ^2^ Slovenian Forestry Institute, Ljubljana, Slovenia; ^3^ Southern Swedish Forest Research Centre, Swedish University of Agricultural Science, Alnarp, Sweden; ^4^ Phytophthora Research Centre, Mendel University in Brno, Brno, Czechia; ^5^ Université de Lorraine, INRAE-Grand-Est, UMR1136 Interactions Arbres, Microorganismes, Nancy, France; ^6^ Swiss Federal Research Institute WSL, Birmensdorf, Switzerland; ^7^ Institute of Forest Ecology Slovak Academy of Sciences, Department of Plant Pathology and Mycology, Nitra, Slovakia; ^8^ Department of Forest Mycology and Plant Pathology, Swedish University of Agricultural Science, Uppsala, Sweden; ^9^ Ukrainian Forest Research Institute, Forestry and Forest Melioration, Kharkiv, Ukraine, Slovakia

**Keywords:** Dothistroma needle blight, *Dothistroma pini*, microsatellites, mating types, pine needle pathogen, *Mycosphaerella pini*, red band needle blight

## Abstract

Dothistroma needle blight (DNB) is an important disease of *Pinus* species that can be caused by one of two distinct but closely related pathogens; *Dothistroma septosporum* and *Dothistroma pini*. *Dothistroma septosporum* has a wide geographic distribution and is relatively well-known. In contrast, *D. pini* is known only from the United States and Europe, and there is a distinct lack of knowledge regarding its population structure and genetic diversity. The recent development of 16 microsatellite markers for *D. pini* provided an opportunity to investigate the diversity, structure, and mode of reproduction for populations collected over a period of 12 years, on eight different hosts in Europe. In total, 345 isolates from Belgium, the Czech Republic, France, Hungary, Romania, Western Russia, Serbia, Slovakia, Slovenia, Spain, Switzerland, and Ukraine were screened using microsatellite and species-specific mating type markers. A total of 109 unique multilocus haplotypes were identified and structure analyses suggested that the populations are influenced by location rather than host species. Populations from France and Spain displayed the highest levels of genetic diversity followed by the population in Ukraine. Both mating types were detected in most countries, with the exception of Hungary, Russia and Slovenia. Evidence for sexual recombination was supported only in the population from Spain. The observed population structure and several shared haplotypes between non-bordering countries provides good evidence that the movement of *D. pini* in Europe has been strongly influenced by human activity in Europe.

## 1 Introduction

Dothistroma needle blight (DNB) is recognized as one of the most important diseases of *Pinus* spp., both in planted and native forests, worldwide. The disease has a long history of having damaged plantations in the Southern Hemisphere dating back to the 1960s (Gibson, 1972), but during the course of the last three decades, it has also increased in severity and incidence in the Northern Hemisphere (Drenkhan and Hanso, 2009; Welsh et al., 2009; Fabre et al., 2012; Boroń et al., 2016; Drenkhan et al., 2016; Ghelardini et al., 2020). Dothistroma needle blight has been reported on 113 taxa, of which 99 are in the genus *Pinus* (Drenkhan et al., 2016; Jánošíková-Hečková et al., 2018; Barnes et al., 2022) and reports of the disease on new hosts and in new geographical regions are increasing (Jánošíková-Hečková et al., 2018; Matsiakh et al., 2018; Mullett et al., 2018; Ondrušková et al., 2018; EPPO, 2019; Mesanza et al., 2021). The disease has been reported on *Abies, Cedrus, Larix, Picea,* and *Pseudotsuga* (Drenkhan et al., 2016), although in most cases, infection has occurred when high inoculum load of the pathogen was present on *Pinus* species in close proximity to these hosts (Barnes et al., 2022).

For many years, the identity of the causal agents of DNB was confused and strongly debated (Barnes et al., 2016). This was due to a single distinct symptom (red bands on infected needles) and taxonomy reliant on morphological characteristics of the associated pathogen. Almost 110 years after the first description of DNB in France (Vuillemin, 1896), it was conclusively shown that two distinct species can cause this disease. These include *Dothistroma septosporum* (Dorogin) M. Morelet and *Dothistroma pini* Hulbary that are most effectively distinguished based on molecular identification (Barnes et al., 2004; Barnes et al., 2016). In an attempt to consolidate existing knowledge, an extensive collaboration of pathologists participating in the DIAROD (Determining Invasiveness And Risk Of *Dothistroma:* DIAROD, COST Action FP1102) project documented, as far as possible, the geographic distribution, hosts and mating type distribution of these two *Dothistroma* species (Drenkhan et al., 2016).

*Dothistroma septosporum* has been the most extensively studied of the two DNB pathogens. This is at least in part due to its accidental introduction into various countries of the Southern Hemisphere where it became one of the most important constraints to plantation forestry based on non-native *Pinus radiata* (Gibson, 1972). *Dothistroma septosporum* has now been recorded in both the Southern and Northern Hemispheres in 48 countries (Drenkhan et al., 2016; Matsiakh et al., 2018; Mullett et al., 2018; Ghelardini et al., 2020) and its population structure and diversity in many of these areas is well understood (Drenkhan et al., 2013; Barnes et al., 2014b; Mullett et al., 2015; Adamson et al., 2018; Oskay et al., 2020; Capron et al., 2021; Mullett et al., 2021). Several genomes of the pathogen have been sequenced and population genomics studies (Ennos et al., 2020), as well as investigations considering factors affecting its pathogenicity have been conducted (Bradshaw et al., 2019; Guo et al., 2020). In contrast, very little is known regarding the biology or ecology of *D. pini*.

*Dothistroma pini* is known only in the Northern Hemisphere where it has been recorded in 17 countries on 19 different *Pinu*s hosts as well as *Picea abies* (Drenkhan et al., 2016; Jánošíková-Hečková et al., 2018; Matsiakh et al., 2018; Mullett et al., 2018; Ondrušková et al., 2018). The pathogen was first described on non-native *Pinus nigra* J.F. Arnold collected in Michigan (1960s), Minnesota and Nebraska in the United States (Barnes et al., 2004). At that time, it was thought to be restricted to the North American continent. Since then, *D. pini* has been reported in four additional states of the United States (Barnes et al., 2014a; Mullett et al., 2018).

*Dothistroma pini* was first discovered in Europe when it was found in the Ukraine and Russia in 2008 on non-native *P. nigra* subsp. *pallasiana* (Lamb.) Holmboe (Barnes et al., 2008b). However, molecular analysis of herbarium samples collected in France have shown that the pathogen has been present on the European continent at least since 1907 (Fabre et al., 2012). Since the first molecular identification of *D. pini* in Europe in 2008, the pathogen has also been confirmed as present in Belgium (Schmitz et al., 2013), Czech Republic (Bergová and Kryštofová, 2014), France (Ioos et al., 2010), Georgia (Matsiakh et al., 2018), Germany (EPPO, 2019), Hungary (Barnes et al., 2011), Montenegro (Lazarević et al., 2017), Poland (Wartalska et al., 2021), Romania (Barnes et al., 2016), Serbia (Pap et al., 2015), Slovenia (Piškur et al., 2013), Slovakia (Ondrušková et al., 2017), Spain (Iturritxa et al., 2015) and Switzerland (Queloz et al., 2014).

Very little is known regarding the genetic diversity and population structure of *D. pini.* In a preliminary study testing 16 microsatellite markers developed for *D. pini* (Siziba et al., 2016), high levels of genetic diversity were found in populations of the pathogen in France, at least indicating the presence of the pathogen in that country for many years. In contrast, populations in other European countries such as Slovakia displayed low genetic diversity and strong signals of clonality, which suggests that *D. pini* was introduced into Slovakia (Adamčíková et al., 2021).

Collections of *D. pini* made over a 12-year period, and including those obtained while documenting the presence of both this species and *D. septosporum* in Europe by the DIAROD cost action, has resulted in a collection of 345 isolates. This collection provided an opportunity to expand on previous, relatively small-scale studies (Siziba et al., 2016; Adamčíková et al., 2021), and to more comprehensively consider the population structure and diversity of *D. pini* in Europe. The aims of this study were thus to 1) investigate the genetic diversity and population structure of the pathogen including countries or specific locations where the pathogen has been reported in Europe, and 2) determine its mode of reproduction and likely means of dispersal in Europe.

## 2 Materials and methods

### 2.1 Sample collection, fungal isolations and identifications

**FIGURE 1:**
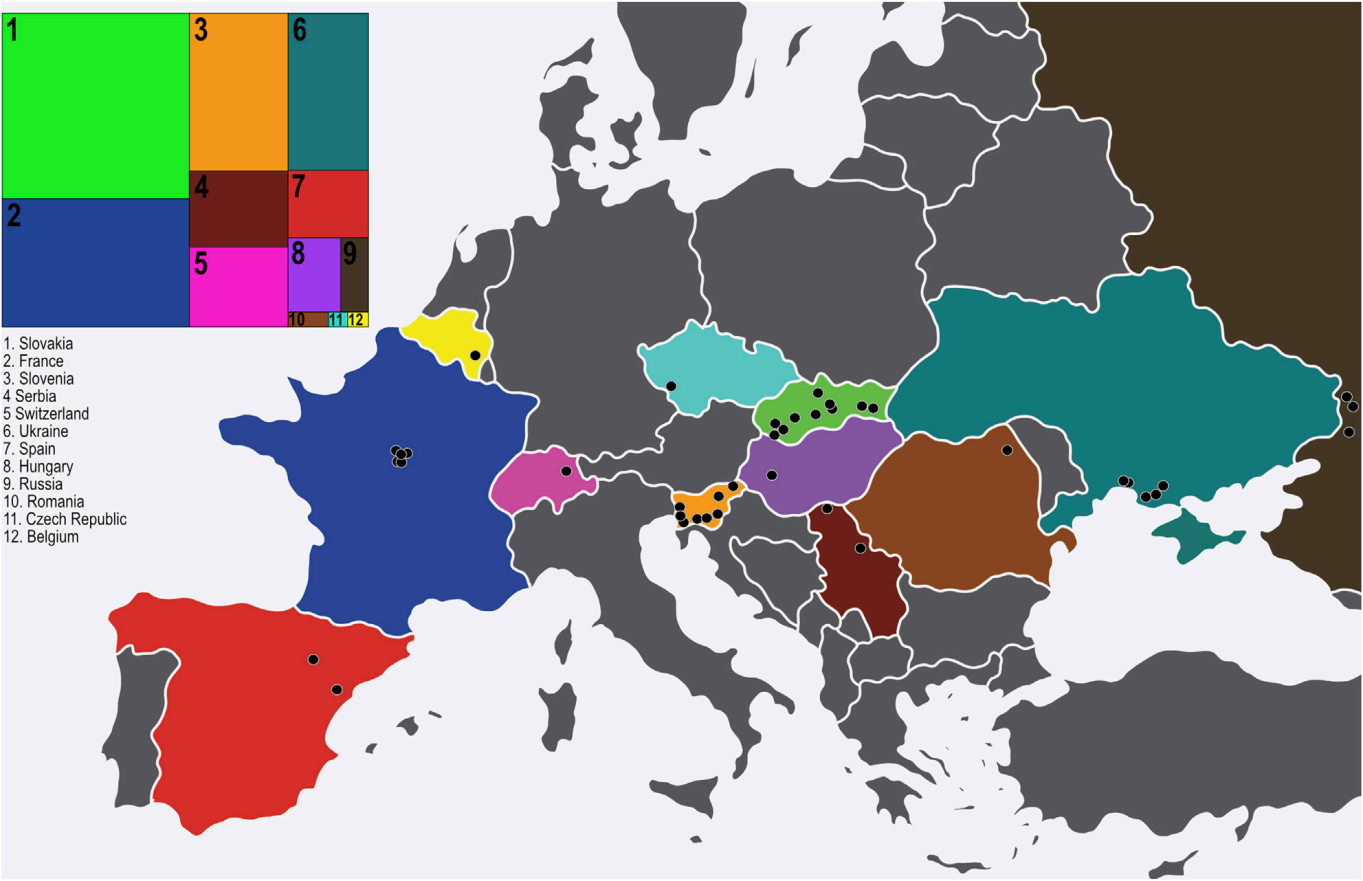
The 40 sampling locations of *D. pini* in Europe. The insert to the left indicates the proportion of isolates obtained per country in relation to other countries (numbered from 1–12). The countries are colour coded on the map to match the insert (original map obtained from https://www.vecteezy.com/free-vector/europe-map) and each sampling location is indicated with a black circle.

Pine needles that displayed DNB symptoms were collected between 2008 and 2019 from 30 locations in 11 countries of Europe (Supplementary Table S2, Figure 1). Additionally, the data generated for the 10 locations in Slovakia by Adamčíková et al. (2021) were incorporated in this study. For most samples, isolations were made from the collected samples as described by Barnes et al. (2004). Single germinating conidia were selected and plated onto 2% Dothistroma Sporulating Media (DSM: 5 g yeast extract (Biolab, Merck, Modderfontein, South Africa), 20 g malt extract (Biolab) and 15 g agar (BD Difco™, Sparks, MD)) per liter of distilled water with 100 mg/l streptomycin (Sigma-Aldrich, St Louis, MO). The plates were incubated for 4–6 weeks at 23°C under natural day/night light cycles. All isolates are either maintained as cultures or freeze-dried material in the culture collection (CMW) of the Forestry and Agricultural Biotechnology Institute (FABI) in Pretoria, South Africa (Supplementary Table S2).

Fungal tissue was freeze dried and DNA extracted using a Zymo Research ZR fungal/Bacterial DNA MiniPrep™ kit (Irvine, CA) as described by van der Nest et al. (2019b). The identity of the isolates was determined by amplifying and sequencing the internal transcribed spacers (ITS) 1 and 2 and the 5.8 S rDNA region with the ITS1 and ITS4 primers (White et al., 1990) and using the protocols described in Barnes et al. (2004). The PCR amplicons were sequenced in both directions using the BigDye Terminator v3.1 Cycle Sequencing Kit (Thermo Fisher Scientific) and the product was run on an ABI PRISM 3500xl capillary auto sequencer (Thermo Fisher Scientific).

CLC Main workbench version 8.0 (CLC Bio, https://www.qiagenbioinformatics.com/products/clc-main-workbench/) was used to create consensus sequences using the forward and reverse sequences of the ITS region for each isolate. All consensus sequences were compared in a BLAST analysis against the GenBank database (NCBI; http://www.ncbi.nlm.nih.gov/genbank/) to confirm the identity of each isolate. To determine the ITS haplotype for each confirmed isolate of *D. pini,* sequences were compared to those reported in Barnes et al. (2016); Mullett et al. (2018) using MEGA 7.0.14 (Kumar et al., 2016).

### 2.2 Microsatellite amplification and haplotype determination

Sixteen labelled microsatellite markers (Siziba et al., 2016) were used to amplify all isolates considered in this study. An additional marker (Doth_A; Barnes et al. (2008a)) was included as an internal diagnostic marker. PCR reactions were performed, and where needed, optimized as described by Adamčíková et al. (2021) (see also Supplementary Table S3) to produce single PCR products. PCR reactions were carried out on an Applied Biosystems^®^ Veriti^®^ 96 well Thermal cycler (Thermo Fisher Scientific, Waltham, MA). The fragments were amplified using the same cycling conditions described by Barnes et al. (2014b) with primer pair annealing temperatures as described by Adamčíková et al. (2021) (see also Supplementary Table S3). To determine amplification success, 5 µl PCR product was stained with 1 µl GelRed nucleic acid gel stain (Biotium), separated by gel electrophoresis on 2% SeaKem LE agarose gel (Lonza) for 15 min at 90 V and visualized under a UV light using a GelDoc EZ Imager (BioRad).

PCR products were pooled in two panels for fragment analysis as described by Siziba et al. (2016) and with adjusted dilutions as indicated in Supplementary Table S3. In preparation for analysis, 1 μl of the pooled product was added to 0.14 μl GENESCAN™ -500 LIZ^®^ (Life Technologies, Applied Biosystems, Warrington, United Kingdom) size standard and 12 μl formamide. Fragment analyses of the prepared reactions was conducted at the University of Pretoria in South Africa with an ABI PRISM 3500xl capillary auto sequencer (Thermo Fisher Scientific). Allele sizes were scored using GENEMAPPER^®^ Software version 5.0 (Applied Biosystems, Foster City, CA).

Alleles scored for each marker were combined to obtain a multilocus haplotype (MLH) for each isolate. Individual isolates were considered clones if they had the same combination of alleles for each marker analyzed. The R package *poppr* (Kamvar et al., 2014) was used to determine the number of MLHs in the dataset. Two datasets were generated for further analyses; the dataset that had not been clone-corrected included all individuals and the clone-corrected dataset contained single representatives of each unique MLH per population. Individuals from each particular country were grouped as populations.

### 2.3 Genetic diversity

The R package *poppr* (Kamvar et al., 2014) was used to calculate the number of MLHs, the expected number of MLHs based on rarefaction (Hurlbert, 1971), the Shannon-Wiener Index (Shannon, 2001), the Stoddart and Taylor’s Index (Stoddart and Taylor, 1988), the Simpson’s Index (Simpson, 1949) and genotypic evenness (Grünwald et al., 2003) for the populations using the non-clone-corrected dataset, as well as the genetic diversity (Nei, 1978) per population using the clone-corrected dataset. The clonal fraction was calculated as in Barnes et al. (2014b). Furthermore, allelic richness (*A*_R_) and private allelic richness (*PA*_R_) were determined using ADZE (Szpiech et al., 2008) that uses rarefaction to allow for comparisons between populations with varying sample sizes. Calculations were standardized corresponding to the country with the smallest population size (Russia, N = 6). A minimum spanning network using Bruvo’s genetic diversity (Bruvo et al., 2004) comparing the MLHs over 16 microsatellite loci was also drawn using the *ismn* function in the *poppr* package.

### 2.4 Population structure

The clone-corrected dataset was used to determine the most likely number of population clusters based on microsatellite allele sizes for all the individuals using STRUCTURE 2.3.4 (Falush et al., 2003). The program assigns individuals to clusters (K) using a Bayesian clustering algorithm. Thirty independent runs of K = 1–20 were performed, with a burn-in value of 100,000 and 500,000 iterations. An admixture model with correlated allele frequencies was selected with no additional priors such as information on the host or location.

The optimal number of clusters was estimated with StructureSelector (Li and Liu, 2018). StructureSelector implements the Evanno method that includes delta (K) and LnP (K) (Evanno et al., 2005) with the additional four Puechmaille methods (MAXMEAK, MAXMEDK, MEDMEDK and MEDMEAK) that provide a more accurate estimate of K in populations with uneven sizes (Puechmaille, 2016). In order to implement the Puechmaille methods, countries were assigned as populations in the dataset and the analysis was repeated twice. First a threshold of 0.5 was selected and second a threshold of 0.8 was selected to apply more stringent assignment of individuals into clusters. After the optimal K was determined, isolates were assigned into the optimal K clusters with a final STRUCTURE run with 30 independent runs, a burn-in value of 100,000 and 1,000 000 iterations. CLUMPAK (Kopelman et al., 2015) was used to converge all 30 runs of the optimal K and the output was visualized using the DISTRUCT program (Rosenberg, 2004). Both CLUMPAK and DISTRUCT were implemented using the StructureSelector website (https://lmme.qdio.ac.cn/StructureSelector/).

The *adegenet* package in R studio (Jombart and Ahmed, 2011) was used to perform discriminant analysis of principal components (DAPC) (Jombart et al., 2010) to additionally visualize the population genetic structure of the European samples. The *find.clusters* function was used to determine the optimal number of clusters by assessment of the Bayesian information criterion (BIC). The optimal number of principal components retained in the analysis was determined by cross-validation using the *xvalDapc* function.

An Analysis of Molecular Variance (AMOVA) test was implemented in GENALEX version 6.5 (Peakall and Smouse, 2012). The test was used to evaluate if there was genetic differentiation among and within groups according to host species, countries and locations. One thousand permutations of the dataset were used to test significance. The null hypothesis of no genetic difference was rejected at *p* < 0.05.

### 2.5 Mating type determination and random mating

The mating type of the *D. pini* isolates was determined by using the primers of Groenewald et al. (2007) or in some cases the primer set of Janoušek et al. (2014). Each reaction consisted of 2 μl template DNA (20 ng/μl concentration), 0.08 μl Faststart Taq DNA polymerase, 0.25 μl of each of the primers as specified by either Groenewald et al. (2007) or Janoušek et al. (2014), 0.6 μl of a mix of 200 mM dNTPs, 1.5 μl of 2.5 mM MgCl_2,_ 1.25 μl 10x PCR reaction buffer and the volume was adjusted to 12.5 μl with sterile SABAX water.

PCR reactions were carried out on an Applied Biosystems^®^ Veriti^®^ 96 well Thermal cycler (Thermo Fisher Scientific, Waltham, MA). The cycling conditions for all microsatellite fragments included an initial denaturation step at 95°C for 4 min, 10 cycles consisting of 94°C for 20 s, a 45 s annealing step with the temperature set according to the protocols by Groenewald et al. (2007) or Janoušek et al. (2014), and an elongation step of 45 s at 72°C. This was followed by a further 25 cycles of 94°C for 20 s, 45 s with a 5 s extension step per cycle at the annealing temperature, a 72°C extension for 45 s and a final extension step of 72°C for 30 min. The amplified products were visualized by staining 10 µl of each product with GelRed™ nucleic acid gel stain. The fragments were separated on 2% SeaKem^®^ LE agarose gel for 50 min at 90 V and viewed under a UV light using the GelDoc™ EZ Imager (BioRad, Hercules, CA). When using the Groenewald et al. (2007) primers, isolates that had an amplicon size of 820 bp were assigned as *MAT1-1* and those with a size of 480 bp were assigned as *MAT1-2*. The Janoušek et al. (2014) primer sets produced amplicon sizes of approximately 560–634 bp for *MAT1-1* and 288–323 bp for *MAT1-2*.

The possibility of sexual recombination was investigated using three methods. An exact binomial test, using two-tailed *p*-values (http://www.biostathandbook.com/exactgof.html) was used to test if the mating type ratios deviated from a 1:1 ratio (at *p* < 0.05) in the non-clone-corrected dataset, which provides evidence of random mating. The index of association (I_A_) (Brown et al., 1980; Smith et al., 1993) and rBarD ( $\bar{r}$ _d_) (Agapow and Burt, 2001) was used to test for linkage disequilibrium in the 16 microsatellite loci with both datasets using the R-package *poppr* (Kamvar et al., 2014). The null hypothesis of alleles at different loci having no linkage due to sexual mating was rejected when *p* < 0.05.

## 3 Results

### 3.1 Sample collection, fungal isolations and identification

A total of 345 cultures included in this study were obtained from collections made in Europe. All of these isolates screened with the Doth_A marker (Siziba et al., 2016) produced an allele size of 111 bp and were thus confirmed as *D. pini.* These included representatives from 12 (Belgium, Czech Republic, France, Hungary, Romania, Western Russia, Serbia, Slovakia, Slovenia, Spain, Switzerland and Ukraine) of the 16 European countries where *D. pini* has been reported. The isolations were made from plant material obtained from 10 different *Pinus* species or sub-species with *P. nigra* being the most common of these (Supplementary Table S1).

Three of the six known *D. pini* ITS haplotypes (Barnes et al., 2016; Mullett et al., 2018) were identified in the collection of isolates (Supplementary Table S1). Individuals having the ITS Haplotype 1 were the most abundant and were present in eight of the twelve countries (Czech Republic, France, Hungary, Slovakia, Slovenia, Spain, Switzerland, Ukraine) including 25 different locations. ITS Haplotype 2 was the second most abundant and was present in eight of the twelve countries (France, Romania, Western Russia, Serbia, Slovakia, Spain, Switzerland, Ukraine) and at 20 different locations. ITS Haplotype 4 individuals were present at nine locations in five countries (Belgium, France, Serbia, Slovakia and Spain). All three haplotypes were present in France, Spain and Slovakia.

### 3.2 Microsatellite amplification and haplotype determination

A total of 109 alleles were detected across the 16 polymorphic microsatellite loci. The number of alleles at each locus ranged from 2 at DP-MS4 and DP-MS18 to 19 at DP-MS12 (Supplementary Table S3). Isolates from Spain, Ukraine and Russia had the highest percentage (87.5%) of polymorphic loci (Supplementary Table S2) and those from Hungary had the lowest percentage (31.2%) of polymorphic loci (excluding countries for which only single isolates were available).

**FIGURE 2:**
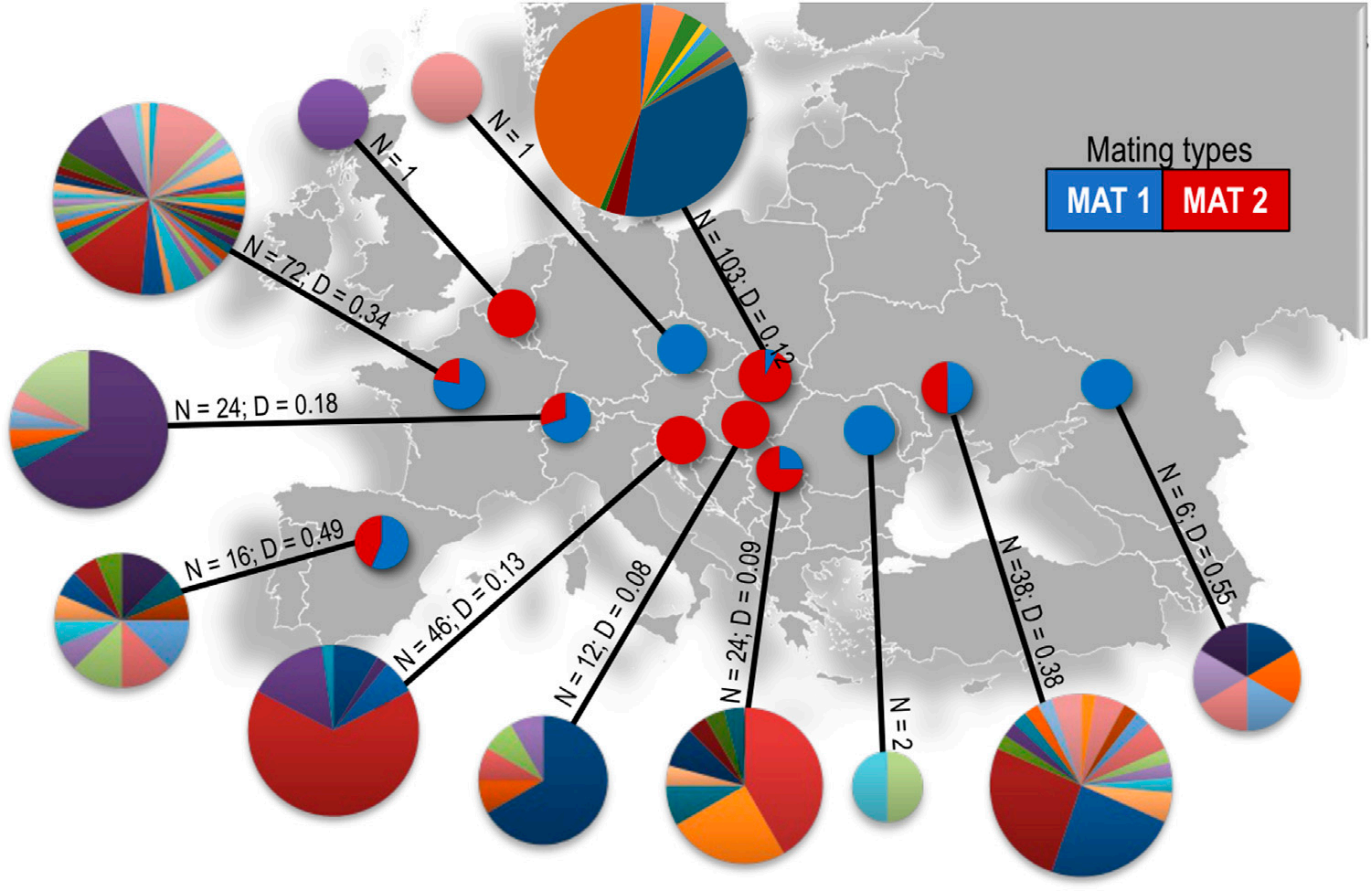
Microsatellite haplotype diversity and mating type ratios of *D. pini* in each of the sampled countries in Europe. Each colour in the pie charts represents a different multilocus haplotype. The size of each MLH pie chart is proportional to the number of isolates per country where Belgium *N* = 1 and Slovakia *N* = 103. *N* = number of isolates, D = Nei’s genetic diversity.

A total of 109 unique multilocus haplotypes (MLHs) were identified in the 345 isolates analyzed (Table 1; Figure 2, Supplementary Table S2) of which eight MLHs occurred in multiple, often non-bordering countries (Supplementary Figure S1). Some individuals sharing the same microsatellite MLH in different populations were of opposite mating type or of different ITS haplotypes, which suggests that they were not true clones. For example, MLH 52 (Supplementary Figure S1) occurred in isolates from four countries (Hungary, Slovakia, Slovenia and Ukraine) and at seven different locations, covering a distance of approximately 1500 km. This MLH was represented by individuals with the *MAT1-1* idiomorph in Ukraine and the *MAT1-2* idiomorph in the other three countries. The fifth most commonly occuring MLH (MLH 83, Supplementary Figure S1) was shared by individuals from the Czech Republic, France (La Bouyale, La Ferté-Imbault, and Villefranche-sur-Cher), and Hola Prystan in Ukraine. All of these individuals were of ITS Haplotype 1, except for an individual from La Ferté-Imbault (ITS Haplotype 4) and the individuals from Hola Prystan in Ukraine (ITS Haplotype 2). Furthermore, all individuals were *MAT1-1*, except for two *MAT1-2* individuals; one individual from La Bouyale in France and one individual from Hola Prystan in Ukraine. The population from Russia included an individual having ITS Haplotype 2 that shared MLH 47 (Supplementary Figure S1) with an ITS Haplotype 1 individual in Hungary (1150 km apart) also of opposite mating types.

**TABLE 1:** Summary diversity statistics of *Dothistroma pini* isolates within populations by country in Europe.

Country^a^	N^b^	MLH^c^	eMLH^d^	CF^e^	Total no of alleles	Unique alleles	A_R_ ^f^	PA_R_ ^g^	H^h^	G^i^	Lambda^j^	E.5^k^	D^l^
Belgium	1	1	N/A	N/A	16	0	N/A	N/A	N/A	N/A	N/A	N/A	N/A
Czech Republic	1	1	N/A	N/A	16	0	N/A	N/A	N/A	N/A	N/A	N/A	N/A
France	72	41	8.52 ± (1.074)	0.43	52	6	1.936 ± (0.215)	0.161 ± (0.054)	3.343	18.51	0.946	0.642	0.344
Hungary	12	5	6.00 ± (0.674)	0.58	23	2	1.236 ± (0.106)	0.066 ± (0.063)	0.674	1.589	0.708	0.623	0.079
Romania	2	2	N/A	N/A	18	1	N/A	N/A	N/A	N/A	N/A	N/A	N/A
Russia	6	6	N/A	0.00	42	1	2.563 ± (0.288)	0.318 ± (0.133)	1.792	6.00	0.833	1.000	0.546
Serbia	24	8	5.58 ± (1.044)	0.67	24	1	1.231 ± (0.090)	0.140 ± (0.070)	1.814	4.36	0.771	0.655	0.087
Slovakia	103	15	4.11 ± (1.149)	0.86	35	5	1.323 ± (0.121)	0.080 ± (0.058)	1.610	3.26	0.693	0.564	0.116
Slovenia	46	6	3.45 ± (0.888)	0.87	30	1	1.361 ± (0.099)	0.079 ± (0.035)	1.122	2.17	0.539	0.564	0.132
Spain	16	12	8.50 ± (0.797)	0.25	59	18	2.562 ± (0.279)	0.701 ± (0.174)	2.426	10.67	0.906	0.937	0.494
Switzerland	24	6	3.57 ± (0.932)	0.75	29	1	1.490 ± (0.142)	0.138 ± (0.076)	1.099	2.09	0.521	0.543	0.184
Ukraine	38	17	6.67 ± (1.257)	0.55	49	3	2.019 ± (0.137)	0.031 ± (0.016)	2.365	6.94	0.856	0.616	0.379
**Total**	**345**	**109**	**8.18 ± (1.181)**	**0.316**	**109**	**39**			**3.724**	**18.00**	**0.944**	**0.420**	**0.425**

^a^ Due to small sample sizes (*N* < 6) in 26/39 of the locations, summary statistics were determined by country.

^b^ N = Total number of isolates.

^c^ Number of multilocus haplotypes. Equivalent to samples that have been clone-corrected.

^d^ The number of expected MLH, at the smallest sample size ≥10 based on rarefaction ± standard error.

^e^ CF: Clonal Fraction = 1—[MLH/N].

^f^ Allelic richness ± standard error (Szpiech et al., 2008). The smallest country sample size considered was 6.

^g^ Privale allelic richness ± standard error (Szpiech et al., 2008). The smallest country sample size considered was 6.

^h^ H: Shannon-Wiener Index of MLH, diversity (Shannon, 2001).

^i^ G: Stoddart and Taylor’s Index of MLH, diversity (Stoddart & Taylor, 1988).

^j^ Lambda: Simpson’s Index (Simpson, 1949) — provides an estimation of the probability that two randomly selected genotypes are different: 0 = no genotypes different. 1 = all genotypes are different.

^k^ E.5: Genotypic evenness, (Grünwald et al., 2003).

^l^ D = Nei’s (1978) gene diversity.

Data in bold indicates the total values for each of the summary statistics.

### 3.3 Genetic diversity

Collections from France had the greatest number of MLHs, followed by the isolates from the Ukraine. When considering populations with a sample size of six and higher, Hungary had the fewest MLHs (five) followed by Russia, Slovenia and Switzerland, which had six each (Table 1). When comparing the approximate number of haplotypes that would be expected for the largest shared sample size (N = 6) based on rarefaction (eMLG), the genotypic richness was the highest in the populations from France and Spain (8.52 and 8.50). The populations from Slovenia and Switzerland had the lowest genetic diversity (3.45 and 3.57 respectively) (Table 1). The Slovenian and Slovakian populations had the highest clonal fractions (0.87 and 0.86) followed by those from Switzerland 0.75 (Table 1). The lowest clonal fraction was found in populations from Russia (0) followed by those from Spain (0.25) and France (0.43). For populations collected within France, the clonal fraction ranged from 0 (Nueng-sur-Beuvron) to 0.61 (Villefranche-sur-Cher). In isolates from Slovenia, the clonal fraction also ranged from 0 (Ribnica) to 0.90 (Panovec). The clonal fraction of 0.55 in Ukraine was due to the high clonal fraction (0.67) in Tsjurupinsk (Supplementary Table S4). The genetic diversity of isolates from all locations is summarized in Supplementary Table S4.

Varying levels of genotypic diversity and genotypic richness were observed for the isolates considered in this study (Table 1). Populations from France followed by Spain displayed the highest level of genetic diversity and richness, based on the Simpson index (H), Stoddart Taylor’s index (G) and allelic richness (A_R_) and rarefaction of MLGs. The genotypic evenness (E.5) observed in the populations from Russia and Spain were the closest to having equal abundance. Using Nei’s unbiased gene diversity, the Russian population had the highest gene diversity (0.546) followed by those from Spain (0.494), Ukraine (0.379) and France (0.344). This could be due to the uneven sample sizes obtained at the different locations because the algorithm does not correct for small population sizes. Populations from Slovenia and Switzerland had the lowest genotypic diversity and genotypic richness. Countries for which only one or two isolates were available (i.e., Romania, Belgium and the Czech Republic) were not considered in the analyses.

The population from Spain had the highest number of private alleles (*PA*_R_) (16.51%) followed by those from France (5.50%) and Slovakia (4.59%). Populations from Russia, Serbia, Slovenia and Switzerland had the lowest number of private alleles (0.90%). Within Slovakia, private alleles were from Arboretum Mlyňany, Jahodná, Košice and Zvolen and in France the private alleles were only from Souesmes (Supplementary Table S4).

### 3.4 Population structure

**FIGURE 3:**
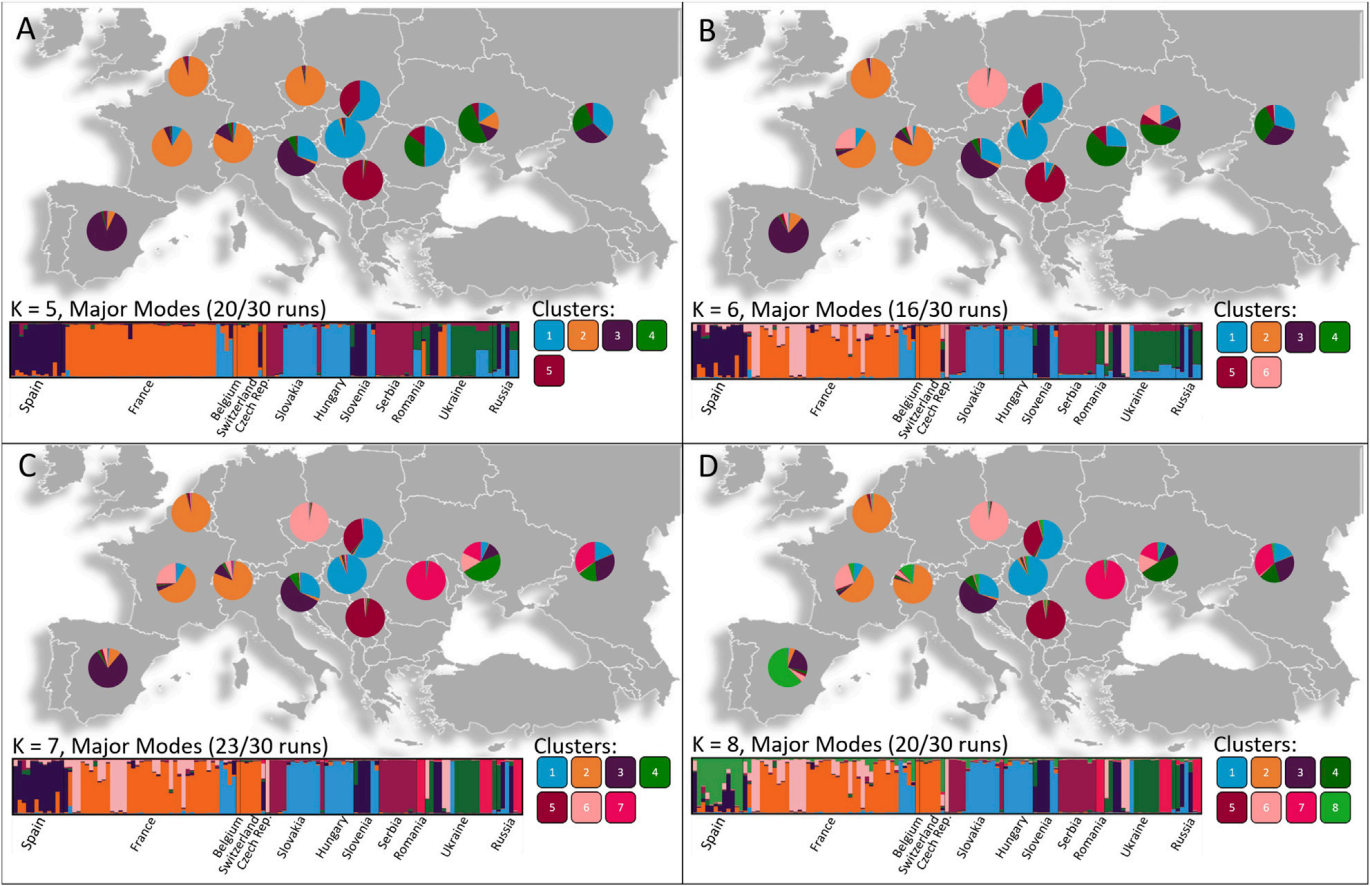
Geographical patterns of population divisions observed using STRUCTURE analyses based on the most likely K values determined by the Puecemaille methods with **(A)** K = 5, **(B)** K = 6, **(C)** K = 7, **(D)** K = 8. The number of individuals belonging to each Cluster is represented as pie charts in each respective country. Four main genetic groups are spread throughout Western, Central and Eastern Europe with several smaller scattered genetic groups residing among the populations.

There was no consensus between different methods of determining the optimal number of clusters in the STRUCTURE analysis. The Evanno ΔK supported nineteen (K = 19) clusters, which indicates that this method failed to detect population structure. LnP (K) suggested K = 10 as the optimal scenario. The four Puechmaille methods suggested that 5–8 clusters are most likely the optimal number of clusters depending on the threshold that was set (Supplementary Figure S2). The STRUCTURE barplots for K = 2 to K = 9 for the major modes are illustrated in Supplementary Figure S3. The barplots for K = 5–8, together with the geographical distribution of the clusters are represented in Figure 3. In order to conduct the DAPC analysis, the *find. clusters* function in the *adegenet* package in R was used and this showed that K resides between 8 and 12. After several runs, K = 10 was proposed as the optimal scenario.

**FIGURE 4:**
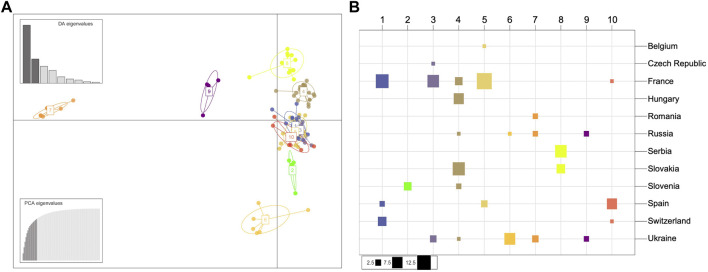
Population structure of the European *Dothistroma pini* collection of isolates. **(A)** Scatterplot of the discriminant analysis of principal components (DAPC) on European *Dothistroma pini* multilocus haplotypes. The number and colours represent the 10 groups delineated by the K-means method. Individual multilocus haplotypes are represented by dots and clusters as ellipses. At the top left the eigenvalues of the first nine axes are represented. **(B)** The composition of the DAPC clusters. The columns and colours correspond to the inferred clusters and the rows correspond to the countries where the populations were sampled. The size of the squares is proportional to the number of individuals comprising each cluster. Cluster one for instance is comprised of individuals isolated from France, Spain and Switzerland with the majority of the individuals in this cluster isolated from France.

For both the K = 8 and K = 10 scenario, the DAPC (Figure 4) and STRUCTURE analysis (Figure 3) indicated that three or four major genetic groups reside between bordering countries in Western, Central, and Eastern Europe. Within these clusters, several smaller genetic groups were observed. The STRUCTURE analysis showed that populations in Western Europe (Belgium, Czech Republic, France and Switzerland) share a major cluster. In Central Europe, one cluster was shared between Hungary, Slovakia and Slovenia and a second genetic cluster was shared between Slovakia and Serbia. In Eastern Europe, isolates from Romania, Russia and Ukraine shared a cluster. Several smaller scattered genetic groups also resided among the populations and the Slovenian population, as well as the Spanish population, included unique genetic clusters.

The DAPC clusters (Figure 4) were mostly correlated with the geographic groups indicated by the STRUCTURE analysis with a Western group containing Cluster 1 (France, Spain, Switzerland), Cluster 3 (Czech Republic, France, Ukraine), Cluster 5 (Belgium, France and Spain) and Cluster 10 (France, Spain and Switzerland). A Central European group accommodated Cluster 4 (France, Hungary, Russia, Slovakia and Slovenia), Cluster 8 (Serbia and Slovakia) as well as a unique cluster (Cluster 2) having only individuals from Slovenia. The DAPC also indicated an Eastern European group with Cluster 6 (Russia, Ukraine), Cluster 7 (Romania, Russia and Ukraine) as well as Cluster 9 (Russia and Ukraine). The four distinct geographic groups suggested by both the STRUCTURE analysis and DAPC were also evident in a haplotype network drawn using Bruvo’s genetic distance (Supplementary Figure S4).

The AMOVA results (Table 2) indicated significant population differentiation according to country (variance among individuals 47%, variance among countries 53%) and even more so by location within countries (variance among individuals 41%, variance among countries 59%). Although this explained less of the variance found among populations, AMOVA also strongly supported the grouping by host species (27% between species and 73% among individuals).

**TABLE 2:** Hierarchical analysis of molecular variance (AMOVA) of *Dothistroma pini* populations, grouped by countries, by locations and by host species.

Source of variation	df	Sum of squares	Mean squares	Estimate of variance	Total variation (%)	*p*-value
Among Countries	8	1,127.55	140.94	1.96	53	0.01
Among Individuals grouped by country	334	1,179.76	3.53	1.77	47	
Within Individuals	343	0.00	0.00	0.00	0	
Total	685	2,307.32		3.72	100	
						
Among Locations	27	1,372.24	50.82	2.05	59	0.01
Among Individuals grouped by location	309	886.36	2.87	1.43	41	
Within Individuals	337	0.00	0.00	0.00	0	
Total	673	2,258.59		3.48	100	
						
Among Hosts	7	507.88	72.55	0.98	27	0.01
Among Individuals grouped by hosts	333	1791.66	5.38	2.69	73	
Within Individuals	341	0.00	0.00	0.00	0	
Total	681	2,299.54		3.67	100	

### 3.5 Mating type determination and random mating

The mating types were successfully amplified for all but two isolates, both from Slovakia (Table 3). Both mating type idiomorphs were detected in isolates from France, Serbia, Slovakia, Spain, Switzerland and Ukraine (Table 3). However, in Nueng-sur-Beuvron in France only *MAT1-1* individuals were detected and in Serbia only *MAT1-2* individuals were present in isolates from Subotica Sands. Similarly, although both mating types were present in the Slovakian collections, either *MAT1-1* or *MAT1-2* individuals were detected at each of the 10 locations sampled in this country. In Ukraine, the population from Nova Zburivka included only one individual that was *MAT1-2* and in Mykolaiv Kinburn, only *MAT1-1* individuals were detected. Although both mating types were found in these countries, random mating was statistically supported only in the populations from Spain, Switzerland and Ukraine as well as in the sub-populations from Souesmes and La Bouyale in France, Deliblato Sands in Serbia, and Hola Prystan, Tsjurupinsk and Mykolaiv Kinburn in Ukraine. In isolates from the Czech Republic, Romania and Russia only *MAT1-1* individuals were present and in those from Belgium, Hungary, and Slovenia only *MAT1-2* individuals were present (Table 3, Supplementary Table S2).

**TABLE 3:** Mating type ratios and index of association tests for the *Dothistroma pini* populations collected in Europe.

Country	Mating type ratios^a^					Linkage disequilibrium—Index of association^b^					
						Non-clone-corrected data			Clone-corrected data		
	*MAT1-1*	*MAT1-2*	Could not determine	Expected ratio	*p*-value (two tailed test)	IA	$\bar{r}$ _d_	*p*-value	I_A_	$\bar{r}$ _d_	*p*-value
Belgium	0	1		N/A	N/A	N/A	N/A	N/A	N/A	N/A	N/A
Czech Republic	1	0		N/A	N/A	N/A	N/A	N/A	N/A	N/A	N/A
France	56	16		36	<0.0001	1.450	0.128	0.0010	0.413	0.036	0.001
Hungary	0	12		6	0.001	−0.055	0.014	0.613	−0.511	0.128	0.970
Romania	2	0		N/A	N/A	N/A	N/A	N/A	N/A	N/A	N/A
Russia	6	0		3	0.031	1.885	0.150	0.001	1.885	0.150	0.002
Serbia	6	18		12	0.023	0.095	**0.019**	**0.281**	−0.265	−0.053	0.818
Slovakia	8	93	2	52	<0.0001	3.685	0.514	0.001	2.492	0.319	0.001
Slovenia	0	46		23	<0.0001	5.265	0.685	0.001	3.151	0.398	0.001
Spain	9	7		**8**	**0.804**	0.974	0.077	0.001	**0.226**	**0.018**	**0.144**
Switzerland	17	7		**10**	**0.115**	5.540	0.794	0.001	4.179	0.604	0.001
Ukraine	19	19		**19**	**1.000**	7.099	0.548	0.001	4.977	0.384	0.001

Statistically non-significant values are highlighted in bold (*p* > 0.05) and indicate random mating is supported by the test.

^a^ Mating type ratios are indicated per country using the non-clone-corrected dataset.

^b^ The index of association tests were conducted per country using both datasets.

Testing linkage disequilibrium using the clone-corrected dataset, with the index of association and rbarD, provided evidence for sexual recombination only in the population from Spain (*p*-value of 0.144). Analysis of the non-clone-corrected dataset also supported evidence of sexual recombination in Serbia (*p*-values of 0.281). This result is however not plausible as the data for both Deliblato Sands and Subotica Sands in Serbia were pooled for this analysis and therefore do not reflect that single mating types were observed at each of these locations.

## 4 Discussion

This study provided the first insights into the population structure and genetic diversity of *D. pini* in Europe. Even though extensive sampling was conducted in the area over a 12-year period, due to the low incidence of *D. pini*, sampling was relatively unstructured and sample sizes were relatively small. This was also emphasized in reports in Switzerland (Dubach et al., 2018) as well as Spain (Ortíz De Urbina et al., 2017) where *D. pini* was less frequently detected than *D. septosporum.* Nonetheless, it was clear that *D. pini* is not new to the European continent and that movement of the pathogen was facilitated through anthropogenic activities.

Based on population structure analyses, the *D. pini* populations considered in this study grouped in four main geographic clusters including one in Western Europe, two in Central Europe, and one in Eastern Europe. Variable population diversity was observed between countries, with France, Spain and Ukraine having the highest levels of genetic diversity and the presence of both mating types. This suggests that *D. pini* has most likely been present in those countries for a long period of time and is in agreement with the identification of *D. pini* in France from herbarium specimens dating back to 1907 and 1965 (Fabre et al., 2012). In contrast, there were populations that were clonal and with a single mating type such as in Slovakia and Slovenia, suggesting more recent introductions. Additionally, the presence of the same MLHs over long distances suggests that human-mediated movement of *D. pini* is taking place in Europe, possibly through plant trade (Pautasso and Jeger, 2014).

Both mating types of *D. pini* were present in many populations considered in this study, but evidence for sexual recombination was supported only in the population from Spain. The fact that some isolates of the same MLHs were of different mating type suggests that sexual recombination could be occurring in other European populations of *D. pini*. This is not unusual and has been found in pathogens such as *Teratosphaeria destructans* (Havenga et al., 2021) as well as *Verticillium dahliae*, a clonally reproducing pathogen, having individuals of opposite mating types that were indicative of cryptic or ancestral sexual recombination events (Milgroom et al., 2014; Short et al., 2014).

*Dothistroma pini* has a limited host range and is currently confined to a particular latitudinal geographical range both in Europe as well as in North America. The majority of the isolates in the present study were from several sub-species of *Pinus nigra* with few collections from *P. coulteri, P. jeffreyi, P. mugo, P. ponderosa, P. schwerinii* and *P. sylvestris.* Many of the single isolates from hosts other than *P. nigra* were from urban areas or arboreta and not from the native ranges of the host trees. This suggests that *D. pini* is most likely not native to the areas where it was collected in Europe and could have been introduced to the continent. This is in contrast to the more commonly occurring *D. septosporum* that is hypothesized to be native to the *P. sylvestris* forests in Northern Europe (Adamson et al., 2018), Eastern Europe and Western Asia (Mullett et al., 2021).

The results of this study have provided no clues to the possible center of origin of the pathogen. The only other area of the world where *D. pini* is known to occur is North America (Barnes et al., 2004; Barnes et al., 2014a; Mullett et al., 2018). Dothistroma needle blight is widespread in the United States and has been reported in 35 states (Drenkhan et al., 2016; Mullett et al., 2018). However, most of the reports were from the time before *D. septosporum* and *D. pini* were conclusively separated based on phylogenetic inference in 2004 (Barnes et al., 2004). Thus, the presence of *D. pini* has been confirmed in only seven states in the Central regions of the United States (Barnes et al., 2004; Barnes et al., 2014a; Mullett et al., 2018) and *D. septosporum* in four states (Barnes et al., 2004; Barnes et al., 2016). The techniques available to discriminate between the two species with relative ease (Barnes et al., 2004; Groenewald et al., 2007; Barnes et al., 2008a; Ioos et al., 2010; Schneider et al., 2019; Aglietti et al., 2021; Myrholm et al., 2021) should simplify efforts to collect isolates known to be those of *D. pini* from the United States, and potentially other unsampled areas such as Asia. This would facilitate an opportunity to compare populations across continents, using either microsatellite markers or whole genome comparisons, in an effort to understand global pathways of spread and potential native areas. The extensive data assembled in the present study will provide a solid foundation for these comparisons.

An intriguing question pertaining to DNB is why *D. septosporum* has spread from the Northern Hemisphere to many Southern Hemisphere countries but that the closely related *D. pini* has not done so. This could be related to host range where *D. septosporum* has mainly been a problem on *P. radiata* in the Southern Hemisphere (Gibson, 1972; Barnes et al., 2014b; Drenkhan et al., 2016), although it has recently emerged as a serious constraint in plantations of *P. tecunumanii* in Colombia (Rodas et al., 2016). Both *Dothistroma* species have relatively wide host ranges and as greater numbers of *Pinus* spp. are being tested and propagated in Southern Hemisphere countries, it seems plausible to suggest that *D. pini* poses an important threat to these resources. Based on experience with *D. septosporum* as well as the increasingly important pine needle pathogen *Lecanosticta acicola* (van der Nest et al., 2019a), and apparently *D. pini* as was found in this study, there is good reason to emphasize the importance of quarantine when moving *Pinus* germplasm between countries and continents.

## Data Availability

The authors acknowledge that the data presented in this study must be deposited and made publicly available in an acceptable repository, prior to publication. Frontiers cannot accept a manuscript that does not adhere to our open data policies.

## Supplementary material

Supplementary Figure S1 The 109 multilocus haplotypes (MLH) derived from microsatellite data in each country and shared between countries. Eight MLHs, indicated by coloured arrows are shared between multiple, often non-bordering countries. France contains the highest number of MLHs, followed by Ukraine. MLH 52 (navy blue) occurred in four countries (Hungary, Slovakia, Slovenia, and Ukraine) in 7 locations, covering a distance of approximately 1500 km. The single individual from the Czech Republic shared MLH 83 (dark green) with isolates from different locations in France and 1480 km away as well as two isolates from Hola Prystan in Ukraine. MLH 56 (purple) was the second most occurring MLH and was shared by individuals in Souesmes (France), Diszel (Hungary) and Arborétum Mlyňany, Gabčikovo, Jahodna and Trstice in Slovakia. MLH47 (red) occurred in the Russian population (ITS Haplotype 2 individual) and 1150 km apart in the Hungarian population (ITS Haplotype 1 individual). MLH 11 (light blue) occurred in the population in Russia as well as in Hola Prystan in Ukraine (620 km apart). MLH 34 (lime green) was present in both Arborétum Mlyňany and Zvolen (Slovakia) as well as Delibratski Pesak and Subotička Pescara (Serbia), while individuals of MLH 32 (yellow) were detected in Kováčová (Slovakia) and Subotička Pescara (Serbia). MLH 59 (orange) was found in both Selles-Saint-Denis and La Ferté-Imbault (France) as well as in the population from Switzerland.

Supplementary Figure S2 The optimum number of clusters determined using STRUCTURESELECTOR. **(A)** The Evanno method (Delta K and LnP (K), suggested K= 19 and K = 10 respectively. When a threshold of 0.5 was set, the Puechmaille methods **(B)** determined the most optimal number of clusters as 7 or 8 and at a threshold of 0.8 **(C)**, the most optimal number of clusters were determined to be 5 or 6.

Supplementary Figure S3 STRUCTURE results of *D. pini* populations per country using the clone-corrected dataset. The structure bar plots show the results for the major clustering modes from K=2 to K=9. The bar plots are divided according to geographical location.

Supplementary Figure S4 Haplotype network of *D. pini* collected in Europe drawn using Bruvo’s genetic distance. Each circle represents a multilocus haplotype. The larger the circle, the more individuals have the same haplotype. The circle size for 1, 22 and 47 individuals of a particular haplotype are indicated on the left hand side for scale. The same four major clusters are observed as with the STRUCTURE analysis with the Western Europe individuals (blue oval), Central European clusters (yellow and pink ovals) and Eastern European cluster (dark green) clustering together.

Supplementary Table S1
*Dothistroma pini* collections from Europe used in this study.

Supplementary Table S2 Allele sizes for 345 individuals of *Dothistroma pini* in Europe based on 17 microsatellite markers.

Supplementary Table S3
*Dothistroma pini* microsatellite PCR annealing temperatures, MgCl2 adjustments, dilutions for fragment analysis for each microsatellite marker and number of alleles per microsatellite marker (Adamčíková et al., 2021).

Supplementary Table S4 Summary diversity statistics of *Dothistroma pini* isolates within locations in Europe.
