# Supplementary figures and images for: Population structure and diversity of the needle pathogen Dothistroma pini suggests human-mediated movement in Europe
Source: Front Genet. 2023 Feb 16;14:1103331. doi: 10.3389/fgene.2023.1103331 (PMC9978111; doi:10.3389/fgene.2023.1103331)

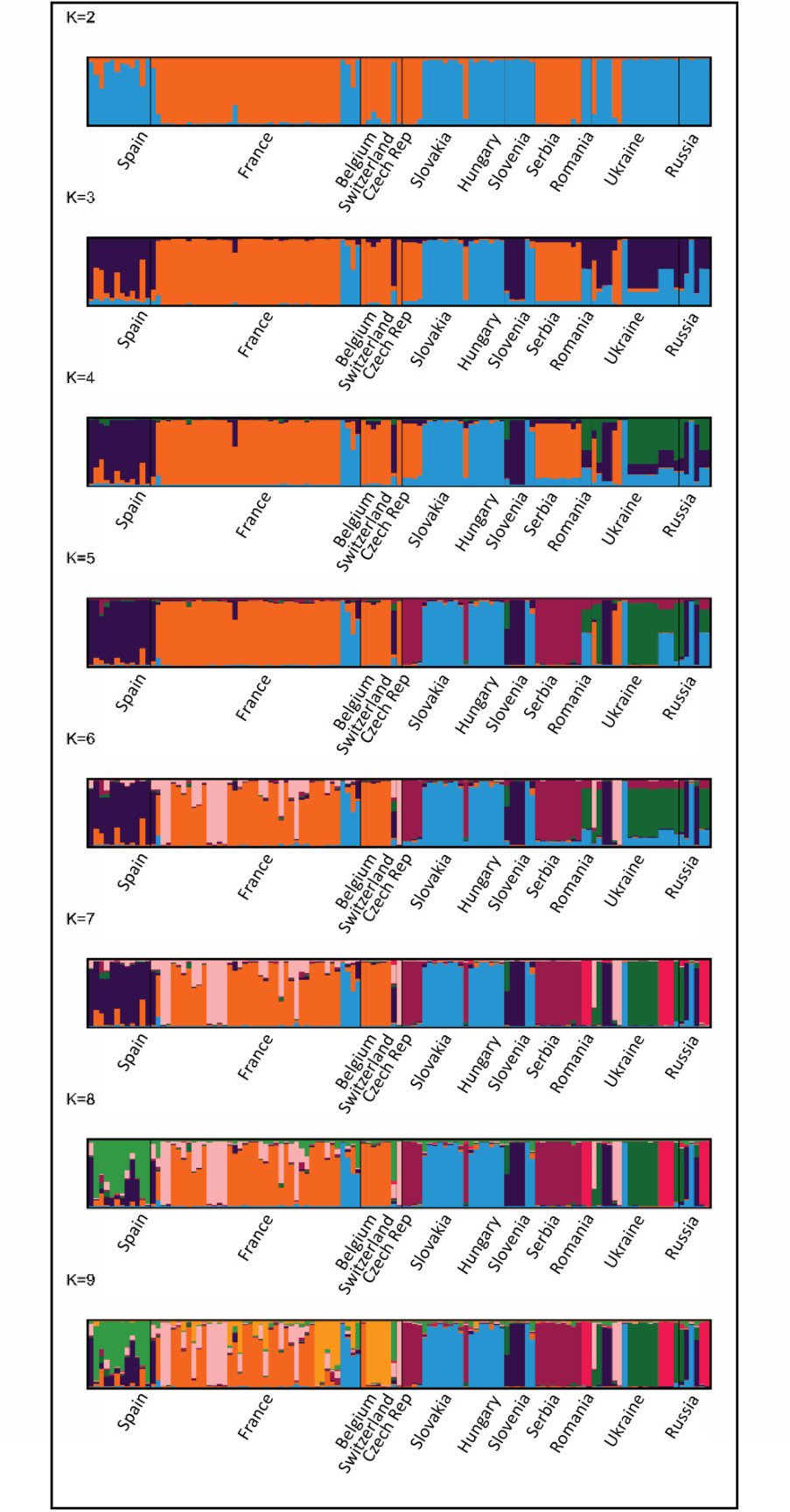

Supplement: Supplementary file 1 [file Image3.tiff]

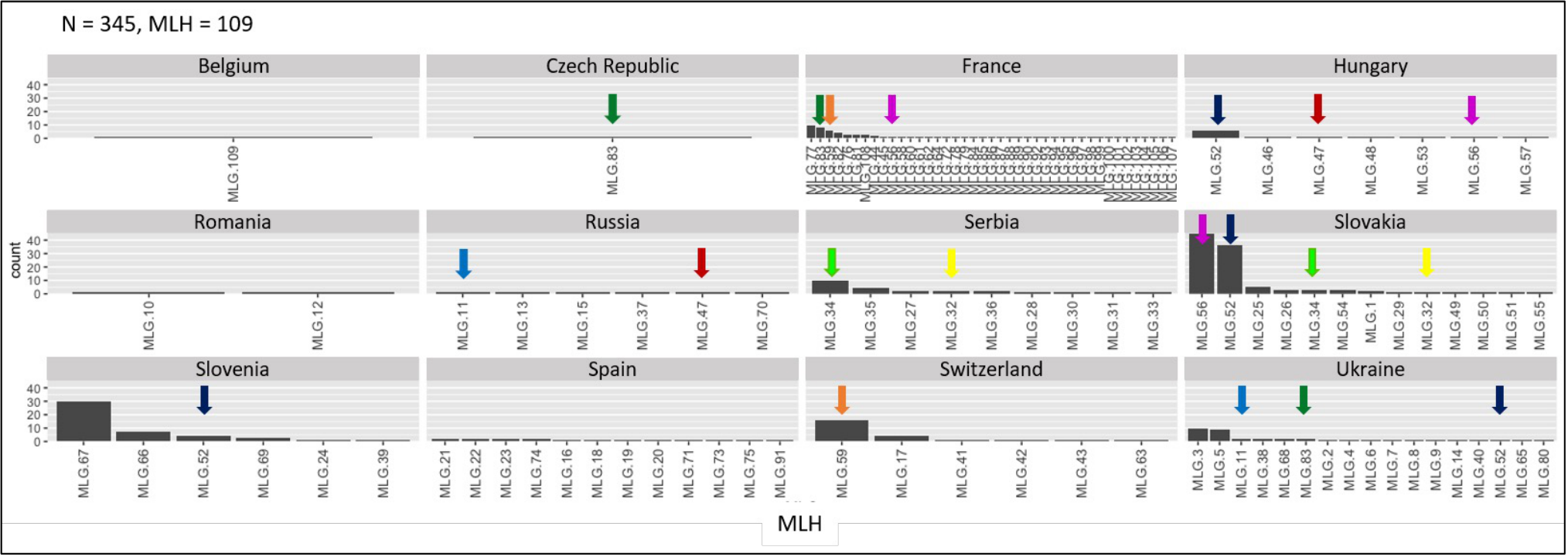

Supplement: Supplementary file 3 [file Image1.tiff]

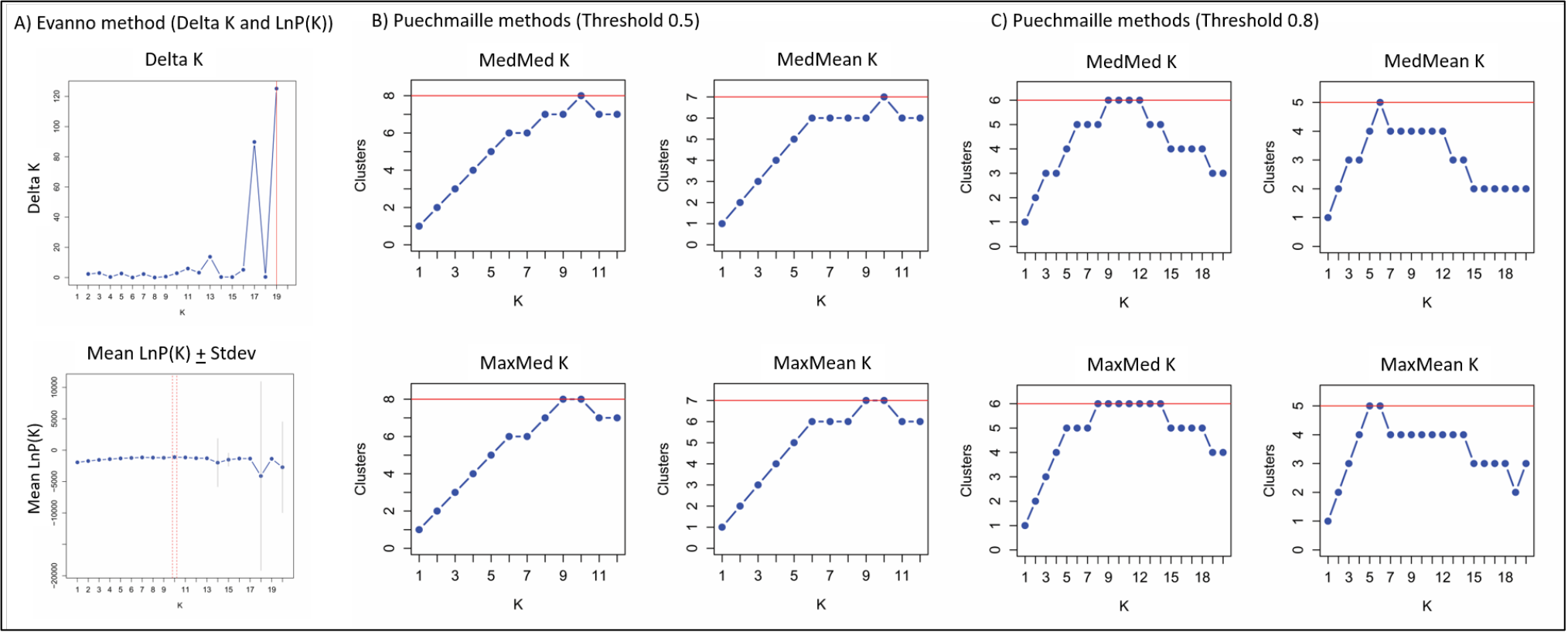

Supplement: Supplementary file 7 [file Image2.tiff]

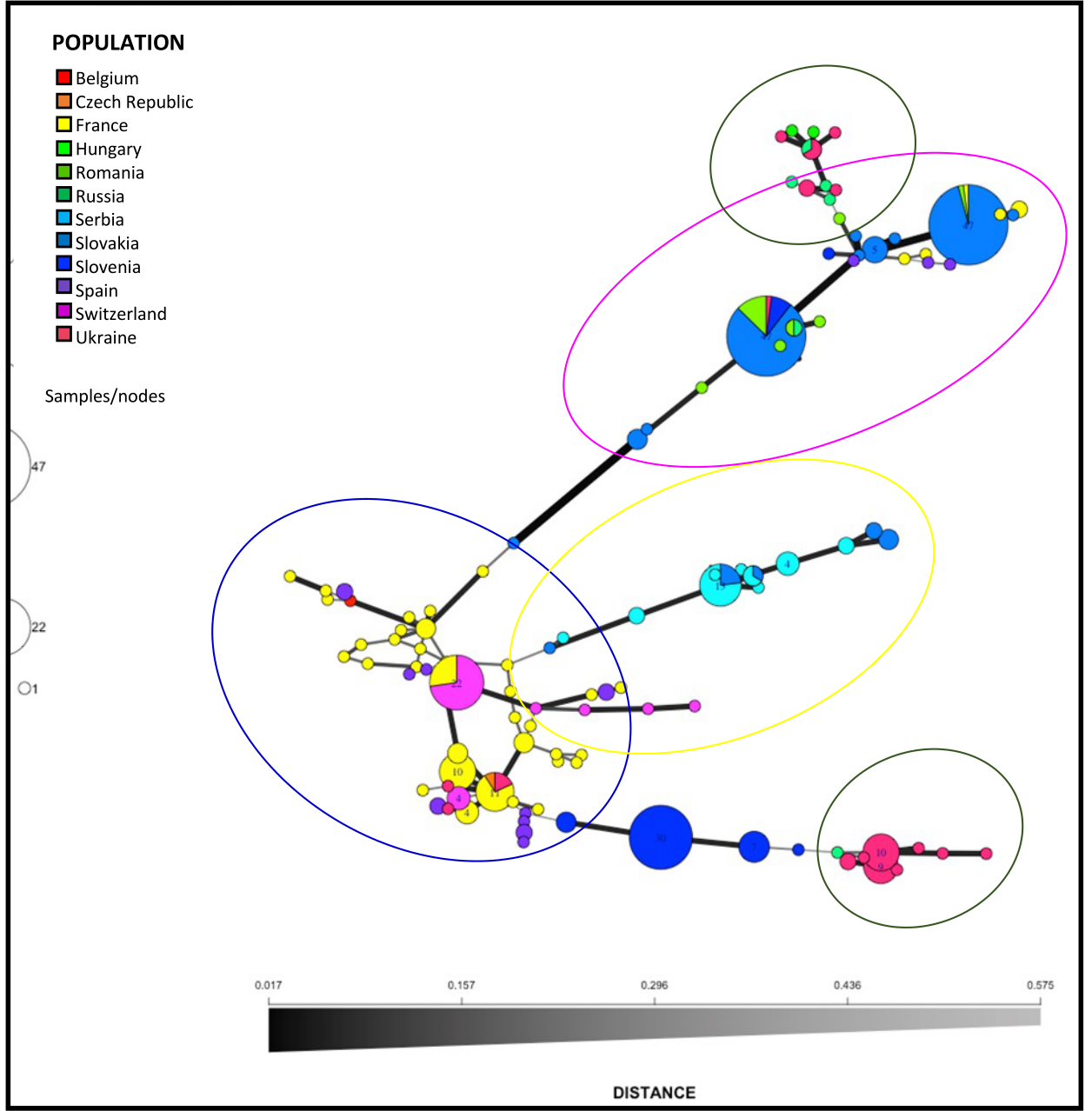

Supplement: Supplementary file 8 [file Image4.tiff]
