# Supplementary material for: Population structure and diversity of the needle pathogen Dothistroma pini suggests human-mediated movement in Europe
Source: Front Genet. 2023 Feb 16;14:1103331. doi: 10.3389/fgene.2023.1103331 (PMC9978111; doi:10.3389/fgene.2023.1103331)
Supplement: Supplementary file 4 [file Table1.docx]

Supplementary Table S1. Dothistroma pini collections from Europe used in this study.

| **Country** | **Isolates per country** | **Isolates per location** | **Locality** | **Collectors** | **Year collected** | **Hosts** | **Plantation type** | **ITS**  **Hap 1/Hap 2/Hap 4** |
| --- | --- | --- | --- | --- | --- | --- | --- | --- |
| **Belgium** | **1** | **1** | **-** | **Mullett MS** | **-** | **-** | **Unknown** | **-/-/1** |
| **Czech Republic** | **1** | **1** | **Bohemia, Chodská Lhota** | **Bergová E** | **2013** | ***P. jeffreyi*** | **Public greenery** | **1/-/-** |
|  |  | 9 | La Bouyale | Barnes I, Mullett MS | 2012 | *P. nigra* subsp. *laricio* | Plantation | 7/2/- |
|  |  | 29 | La Ferté-Imbault | Barnes I, Mullett MS | 2012 | *P. nigra* subsp. *laricio* | Urban greenery | 22/3/3 (1 missing data) |
|  |  | 2 | Neung-sur-Beuvron | Barnes I, Mullett MS | 2012 | *P. nigra* subsp. *laricio* | Plantation | -/-/2 |
|  |  | 14 | Souesmes | Barnes I, Mullett MS | 2012 | *P. nigra* subsp. *laricio* | Plantation | 14/-/- |
|  |  | 18 | Villefranche-sur-Cher | Barnes I, Mullett MS | 2012 | *P. nigra* subsp. *laricio* | Plantation | 17/-/1 |
| **France** | **72** |  |  |  |  |  |  | **60/5/6** (1 missing data) |
| **Hungary** | **12** | **12** | **Diszel** | **Barnes I** | **2007** | ***P. nigra*** | **Pine stand next to road** | **12/-/-** |
| **Romania** | **2** | **2** | **Botoșani** | **Costache C** | **2015** | ***P. nigra*** | **Unknown** | **-/2/-** |
|  |  | 1 | Kamensky district, Rostov oblast | Bulgakov TS | 2006 | *P. pallasiana* | Forest plantation | -/1/- |
|  |  | 3 | Krasnosulinsky district, Donskoye forestry | Bulgakov TS | 2007 | *P. pallasiana, P. mugo, P. nigra* | Forest plantation | -/3/- |
|  |  | 2 | Tarasovsky district, Gorodishchenskoye forestry | Bulgakov TS | 2007 | *P. pallasiana* | Forest plantation | -/2/- |
| **Russia** | **6** |  |  |  |  |  |  | **-/6/-** |
|  |  | 17 | Deliblato Sands, Susara | Keča N | 2014 | *P. nigra* | Plantation | -/6/11 |
|  |  | 7 | Subotica Sands | Sadiković D | 2014 | *P. nigra* | Plantation | -/2/5 |
| **Serbia** | **24** |  |  |  |  |  |  | **-/8/15** |
|  |  | 41 | Arboretum Mlynany | Adamčíková K, Ondrušková E, Hečková Z | 2014, 2015, 2017 | *P. coulteri, P. jeffreyi, P. nigra, P. ponderosa, P. schwerinii* | Arboretum | 37/2/2 |
|  |  | 3 | Banská Belá | Adamčíková K, Ondrušková E | 2016 | *P. nigra* | Urban greenery | -/3/- |
|  |  | 6 | Gabčíkovo | Hečková Z, Adamčíková K | 2013, 2015, 2017 | *P. nigra* | Forest plantation | 6/-/- |
|  |  | 25 | Jahodná | Hečková Z, Adamčíková K | 2013, 2014, 2017 | *P. nigra* | Forest plantation | 25/-/- |
|  |  | 5 | Košice | Adamčíková K | 2015 | *P. ponderosa* | Arboretum | 5/-/- |
|  |  | 1 | Kováčová | Adamčíková K, Ondrušková E | 2017 | *P. mugo* | Urban greenery | -/1/- |
|  |  | 1 | Ľubochňa | Adamčíková K, Ondrušková E | 2014 | *P. sylvestris* | Natural regeneration | 1/-/- |
|  |  | 1 | Sečovce | Adamčíková K | 2015 | *P. mugo* | Urban greenery | -/1/- |
|  |  | 17 | Trstice | Adamčíková K | 2015, 2017 | *P. nigra* | Plantation/nursery | 17/-/- |
|  |  | 3 | Zvolen | Adamčíková K | 2015, 2017 | *P. jeffreyi* | Urban greenery | 2/-/1 |
| **Slovakia** | **103** |  |  |  |  |  |  | **93/7/3** |
|  |  | 2 | Dutovlje (Karst) | Jurc D, Hauptman T | 2013 | *P. nigra* | Unknown | 2/-/- |
|  |  | 2 | Hruševica (Karst) | Jurc D, Hauptman T | 2013 | *P. nigra* | Unknown | 2/-/- |
|  |  | 29 | Panovec | Piškur B, Jurc D | 2013 | *P. nigra* | Unknown | 29/-/- |
|  |  | 4 | Pivka | Sadiković D, Hauptman T | 2013 | *P. nigra* | Natural regeneration | 4/-/- |
|  |  | 4 | Prebold | Jurc D | 2013 | *P. nigra* | Unknown | 4/-/- |
|  |  | 1 | Radenci | Hauptman T | 2015 | *P. nigra* | Unknown | 1/-/- |
|  |  | 2 | Ribnica | Piškur B, Jurc D | 2013 | *P. nigra* | Unknown | 2/-/- |
|  |  | 2 | Škocjan | Jurc D, Hauptman T | 2013 | *P. nigra* | Unknown | 2/-/- |
| **Slovenia** | **46** |  |  |  |  |  |  | **44/-/-** |
|  |  | 15 | Aragon | Mullett MS | 2017 | *P. nigra* subsp*. nigra* | Plantation | 9/2/4 |
|  |  | 1 | Boixar | Mullett MS | 2017 | *P. nigra* subsp. *nigra* | Plantation | -/1/- |
| **Spain** | **16** |  |  |  |  |  |  | **9/3/4** |
| **Switzerland** | **24** | **24** | **Weesen, Walensee** | **Holdenrieder O** | **2012/2013** | ***P. nigra*** |  | **7/17/-** |
|  |  | 9 | Kherson, Hola prystan | Davydenko K | 2013 | *P. nigra* subsp*. pallasiana* | Forest plantation | -/9/- |
|  |  | 1 | Kherson, Nova Zburivka | Davydenko K | 2013 | *P. nigra* subsp*. pallasiana* | Forest plantation | -/1/- |
|  |  | 21 | Kherson, Tsjurupinsk | Usichenko AC, Davydenko K | 2013 | *P. nigra* subsp*. pallasiana* | Forest plantation | 3/18/- |
|  |  | 5 | Mykolaiv Kinburn | Davydenko K | 2013 | *P. nigra* subsp*. pallasiana* | Forest plantation | -/5/- |
|  |  | 2 | Kinburg Peninsula | Davydenko K | 2013 | *P. nigra* subsp*. pallasiana* | Forest plantation | 1/1/- |
| **Ukraine** | **38** |  |  |  |  |  |  | **4/34/-** |
| **Total:** | **345** |  |  |  |  |  |  |  |
