# Supplementary material for: Population structure and diversity of the needle pathogen Dothistroma pini suggests human-mediated movement in Europe
Source: Front Genet. 2023 Feb 16;14:1103331. doi: 10.3389/fgene.2023.1103331 (PMC9978111; doi:10.3389/fgene.2023.1103331)
Supplement: Supplementary file 5 [file Table3.docx]

Supplementary Table S2. Dothistroma pini microsatellite PCR annealing temperatures, MgCl_2_ adjustments, dilutions for fragment analysis for each microsatellite marker and number of alleles per microsatellite marker (Adamčíková et al., 2021).

| **Marker name** | **Optimum annealing temp (°C)** | **Reduced MgCl_2_ for PCR amplification^2^** | **Dilution ratio (μl) for fragment analyses** | **Panel number in fragment analyses** | **Number of alleles^1^ per microsatellite marker** |
| --- | --- | --- | --- | --- | --- |
| Doth_A | 57 | Yes | 2/100 | 1 | 1 |
| DP-MS1 | 62 | Yes | 2/100 | 1 | 13 |
| DP-MS2 | 57 | Yes | 2/100 | 1 | 3 |
| DP-MS4 | 60 | No | 1.5/100 | 1 | 2 |
| DP-MS5 | 58 | Yes | 1/100 | 1 | 6 |
| DP-MS6 | 58 | No | 1.6/200 | 2 | 4 |
| DP-MS7 | 62 | No | 1.6/200 | 2 | 10 |
| DP-MS8 | 60 | Yes | 2/200 | 2 | 4 |
| DP-MS9 | 60 | Yes | 1.4/200 | 2 | 6 |
| DP-MS10 | 58 | Yes | 0.8/100 | 1 | 3 |
| DP-MS11 | 58 | Yes | 1/100 | 1 | 12 |
| DP-MS12 | 60 | Yes | 2/200 | 2 | 19 |
| DP-MS13 | 59 | Yes | 2/100 | 1 | 13 |
| DP-MS15 | 60 | No | 2/200 | 2 | 4 |
| DP-MS16 | 60 | Yes | 1.4/200 | 2 | 3 |
| DP-MS17 | 60 | No | 1.2/200 | 2 | 6 |
| DP-MS18 | 59 | Yes | 1.2/200 | 2 | 2 |

^1^Determined from 345 isolates analysed in Europe.

^2^The total MgCl_2_ was reduced from 1.5ul to 0.9ul in each PCR reaction if required
