# Supplementary material for: Population structure and diversity of the needle pathogen Dothistroma pini suggests human-mediated movement in Europe
Source: Front Genet. 2023 Feb 16;14:1103331. doi: 10.3389/fgene.2023.1103331 (PMC9978111; doi:10.3389/fgene.2023.1103331)
Supplement: Supplementary file 6 [file Table4.docx]

Supplementary Table S3. Summary diversity statistics of *Dothistroma pini* isolates within locations in Europe.

| **Country** | **Location** | **N^1^** | **MLH^2^** | **CF^3^** | **Total no. of alleles** | **Unique alleles** |
| --- | --- | --- | --- | --- | --- | --- |
| **Belgium** | Unknown | **1** | **1** | **N/A** | **16** | **0** |
| **Czech Republic** | Chodská Lhota | **1** | **1** | **N/A** | **16** | **0** |
|  | La Bouyale | 9 | 4 | 0.56 | 25 | 0 |
|  | La Ferté-Imbault | 29 | 25 | 0.14 | 41 | 0 |
|  | Nueng-sur-Beuvron | 2 | 2 | 0.00 | 23 | 0 |
|  | Souesmes | 14 | 12 | 0.14 | 41 | 5 |
|  | Villefranche-sur-Cher | 18 | 7 | 0.61 | 32 | 0 |
| **France** |  | **72** | **41** | **0.43** | **52** | **6** |
| **Hungary** | Diszel | **12** | **5** | **0.58** | **23** | **2** |
| **Romania** | Botasani | **2** | **2** | **0.00** | **18** | **1** |
|  | Kamesky district | 1 | 1 | N/A | 16 | 0 |
|  | Krasnosulinsky district | 3 | 3 | 0.00 | 27 | 1 |
|  | Tarasovsky district | 2 | 2 | 0.00 | 28 | 0 |
| **Russia** |  | **6** | **6** | **0.00** | **42** | **1** |
|  | Deliblato Sands, Susara | 17 | 6 | 0.65 | 24 | 1 |
|  | Subotica Sands | 7 | 3 | 0.57 | 18 | 0 |
| **Serbia** |  | **24** | **8** | **0.67** | **24** | **1** |
|  | Arboretum Mlynany | 41 | 6 | 0.85 | 28 | 2 |
|  | Banská Belá | 3 | 1 | 0.67 | 16 | 0 |
|  | Gabčíkovo | 6 | 2 | 0.67 | 17 | 0 |
|  | Jahodná | 25 | 4 | 0.81 | 19 | 1 |
|  | Košice | 5 | 1 | 0.80 | 16 | 1 |
|  | Kováčová | 1 | 1 | N/A | 16 | 0 |
|  | Ľubochňa | 1 | 1 | N/A | 16 | 0 |
|  | Sečovce | 1 | 1 | N/A | 16 | 0 |
|  | Trstice | 17 | 2 | 0.88 | 17 | 0 |
|  | Zvolen | 3 | 2 | 0.25 | 22 | 0 |
| **Slovakia** |  | **103** | **15** | **0.86** | **35** | **5** |
|  | Dutovlje (Karst) | 2 | 1 | 0.50 | 16 | 0 |
|  | Hruševica (Karst) | 2 | 1 | 0.50 | 16 | 0 |
|  | Panovec | 29 | 3 | 0.90 | 18 | 0 |
|  | Pivka | 4 | 3 | 0.25 | 21 | 0 |
|  | Radenci | 1 | 1 | N/A | 16 | 1 |
|  | Prebold | 4 | 1 | 0.75 | 16 | 0 |
|  | Ribnica | 2 | 2 | 0.00 | 18 | 0 |
|  | Škocjan | 2 | 1 | 0.50 | 16 | 0 |
| **Slovenia** |  | **46** | **6** | **0.87** | **30** | **1** |
|  | Aragon | 15 | 11 | 0.27 | 53 | 12 |
|  | Boixar | 1 | 1 | N/A | 16 | 5 |
| **Spain** |  | **16** | **12** | **0.25** | **59** | **18** |
| **Switzerland** | Walensee | **24** | **6** | **0.75** | **29** | **1** |
|  | Kherson, Hola prystan | 9 | 7 | 0.22 | 38 | 1 |
|  | Kherson, Nova Zburivka | 1 | 1 | N/A | 16 | 0 |
|  | Kherson, Tsjurupinsk | 21 | 7 | 0.67 | 31 | 0 |
|  | Mykolaiv Kinburn | 5 | 4 | 0.20 | 38 | 2 |
|  | Kinburg Peninsula | 2 | 2 | 0.00 | 22 | 0 |
| **Ukraine** |  | **38** | **17** | **0.55** | **49** | **3** |
| **Total** |  | **345** | **109** | **0.316** | **109** | **39** |

Grey highlights with bold text represent the totals per country. Due to small sample sizes (N<6) in 26/39 of the locations, summary statistics were determined by country and are summarized in Table 1.

^1^N = Total number of isolates per location.

^2^Number of multilocus haplotypes. Equivalent to samples that have been clone-corrected.

^3^CF: Clonal Fraction = 1 – [MLH/N].
